# Maintenance Fluids for Late Preterm and Term Infants: Is it Time to Reconsider?

**DOI:** 10.1542/pedsos.2024-000372

**Published:** 2025-05-16

**Authors:** Amanda J. Chang, Daniel J. York, Wenya Chen, Kaeli N. Heidenreich, Malika D. Shah

**Affiliations:** 1 Northwestern University Feinberg School of Medicine, Chicago, Illinois; 2 Department of Neonatology, University of Utah Health, Salt Lake City, Utah; 3 Department of Pediatrics Ann and Robert H. Lurie Children’s Hospital of Chicago, Chicago, Illinois; 4 Prentice Newborn Nursery, Northwestern Memorial Hospital, Chicago, Illinois.

## Abstract

**BACKGROUND:**

Late preterm and term infants represent the majority of neonatal intensive care unit admissions globally, yet their fluid management remains underexplored.

**METHODS:**

We conducted a retrospective medical record review of 174 infants 34 weeks’ gestational age or older who received dextrose-containing fluids shortly after birth. These infants had 24-hour serum sodium measurements at our institution between April 2018 and April 2021. We used regression models to analyze the correlation among intravenous fluid (IVF) intake per kilogram, gestational age, fluid balance (FB), weight change, and sodium status, adjusting for clinical factors.

**RESULTS:**

At 24 hours, the average IVF intake was 57.2 mL/kg/d (SD 14.9). Of the infants, 130 (75%) had positive FB, 128 (74%) maintained or gained weight, 41 (24%) had sodium levels 132 mEq/L or less, and 68 (39%) had sodium 134 mEq/L or less. Positive FB was associated with weight gain and an increased likelihood of hyponatremia. Regression analysis showed a 0.07-mEq/L decrease in serum sodium (95% CI, −0.09 to −0.05; *P* < .001) for every milliliter per kilogram of positive FB and a 6% increase in the odds of sodium 132 mEq/L or less (95% CI, 1.03–1.08; *P* < .001). Term infants exhibited greater decreases in sodium levels than preterm infants. Infants who did not receive enteral feeds had more pronounced sodium decreases compared with those who were fed.

**CONCLUSION:**

Positive FB was common and strongly associated with hyponatremia in infants receiving standard IVF rates. These effects were most significant in term and unfed infants. Current fluid strategies may overestimate needs, particularly for term infants not receiving enteral feeds.

## Introduction

Newborns are particularly susceptible to free water and electrolyte aberrancies because of renal immaturity and insensible water losses,^1^ and these predispositions increase with lower gestational age and birth weight.^2^ Neonates admitted to the neonatal intensive care unit (NICU) are often unable to tolerate full enteral feeds and require intravenous fluids (IVF). Traditional guidelines for IVF therapy in children originated from the study by Holliday and Segar and recommended initiating hypotonic IVF based on the sodium content of breast milk.^3^ Recommendations for amounts of IVF to administer are based on estimated sensible and insensible water losses and are meant to allow for natural physiologic volume contraction, diuresis, and weight loss as neonates transition to the extrauterine environment.^4,5^ Many current unit protocols recommend a fluid strategy based on milliliters per kilogram and day of life for neonates.^6^ For late preterm and term infants, this is typically 60 ml/kg/d of dextrose-containing fluid for the first day of life, followed by an increase to 80 ml/kg/d with the addition of electrolytes on the second day of life.^7-10^

However, data are emerging on using a more dynamic fluid administration strategy for infants of lower gestational ages. In the European Consensus Guidelines on the Management of Respiratory Distress Syndrome in Neonates, the authors recommend tailoring fluids to individual infants with respiratory distress syndrome according to their serum sodium levels, urine output, and weight loss.^11^ Similar data on the efficacy of various fluid administration strategies in near-term and term infants are lacking, despite these infants representing the majority of NICU admissions in the United States,^12^ Ireland and the United Kingdom,^13^ Asia,^14^ and the Middle East.^15^ In a Cochrane review done by Gupta et al, the authors found an uncertainty of evidence regarding the effects of a restricted fluid strategy as compared with a standard fluid strategy in the management of transient tachypnea of the newborn in term and near-term neonates and suggested that additional observational studies are needed.^9^

Furthermore, in the pediatric population, studies have demonstrated a significant association between hypotonic fluids and iatrogenic hyponatremia.^16-18^ Current American Academy of Pediatrics guidelines do not recommend hypotonic fluids in children aged 28 days to 18 years requiring maintenance IVF^19^ but do not extend this recommendation to neonates because similar studies in newborns are lacking. Similarly, in Europe, guidelines recommend initiation of isotonic fluids in critically ill children and regular monitoring of fluid balance (FB) and electrolytes, but there is a large amount of variability in clinical practices surrounding IVF therapy and a need for further research in this field to support evidence-based guidelines.^20,21^ Here, in a retrospective single-center cohort of neonates admitted to a large metropolitan level III NICU, we evaluate current fluid initiation strategies and the associations with serum sodium, urine output, and weight gain and FB at 24 hours of life in late preterm and term infants.

## Methods

### Patient Selection

We reviewed the consecutive records of all infants older than 34 weeks’ gestational age admitted to our large metropolitan level III NICU between April 1, 2018 and April 1, 2021, who received an electronic medical record order of dextrose-containing fluids. We excluded the following infants: infants transferred to our partner level IV hospital for known congenital anomaly, infants who did not receive IVF despite the order, infants with no recorded weight at 24 hours, infants with no recorded sodium at 24 hours, infants without strict urine output recorded, infants who received IVF with electrolytes before 24 hours, and infants who died before 24 hours of life. The study was approved by and performed under a waiver of consent by our institution’s Institutional Review Board (STU00214976) and with the approval of our institution’s Operations and Research Review Committee. The study was performed in accordance with the principles of the Declaration of Helsinki.

### Data Abstraction

Demographic and clinical data, including gestational age, type of delivery, reason for admission, need for and type of respiratory support, amount and type of IVF received, birth and daily weights, urine output, serum sodium levels, and length of stay, were retrospectively collected from hospital records. Amount of IVF and enteral feeds received, urine output, and weights were recorded daily for the first postnatal week. Data were kept in Research Electronic Data Capture (REDCap) in accordance with institutional policy.

### Statistical Analysis

After abstraction of clinical data, the amount of IVF received in the first 24 hours was adjusted for weight as milliliters per kilogram per day, and urine output was recorded as milliliters per kilogram per hour. Percentage of weight change was calculated as (daily weight – birthweight) ÷ birthweight × 100. FB was calculated as (IVF + enteral feeds – urine output) ÷ birthweight in the first 24 hours. Given variation in the definition of clinically significant hyponatremia in this population, 24-hour sodium levels were evaluated using 2 thresholds: ≤134 mEq/L and ≤132 mEq/L.

The Shapiro-Wilk test was used to assess the normality of data. Patient demographics and clinical characteristics were summarized using frequencies and percentages for categorical variables, means with SDs for normally distributed variables, and medians with IQRs for nonnormally distributed variables. Univariable analysis was performed to evaluate associations between selected patient characteristics and dichotomized gestational age (term vs preterm). We used the 2-sample t-test or Wilcoxon rank-sum test for continuous variables and chisquare test of independence for categorical variables.

Univariable and multiple regression analyses were performed to explore the relationships among term vs preterm status, FB, weight change, and serum sodium levels at 24 hours. The multiple regression models were further adjusted for mode of delivery, antibiotic use, and the need for respiratory support. 95% CIs were reported, and a *P* value <.05 (2-sided) was considered statistically significant. All statistical analysis was conducted in R version 4.1.0 within RStudio version 1.2.1335.

## Results

### Infant Demographics

**FIGURE 1.**
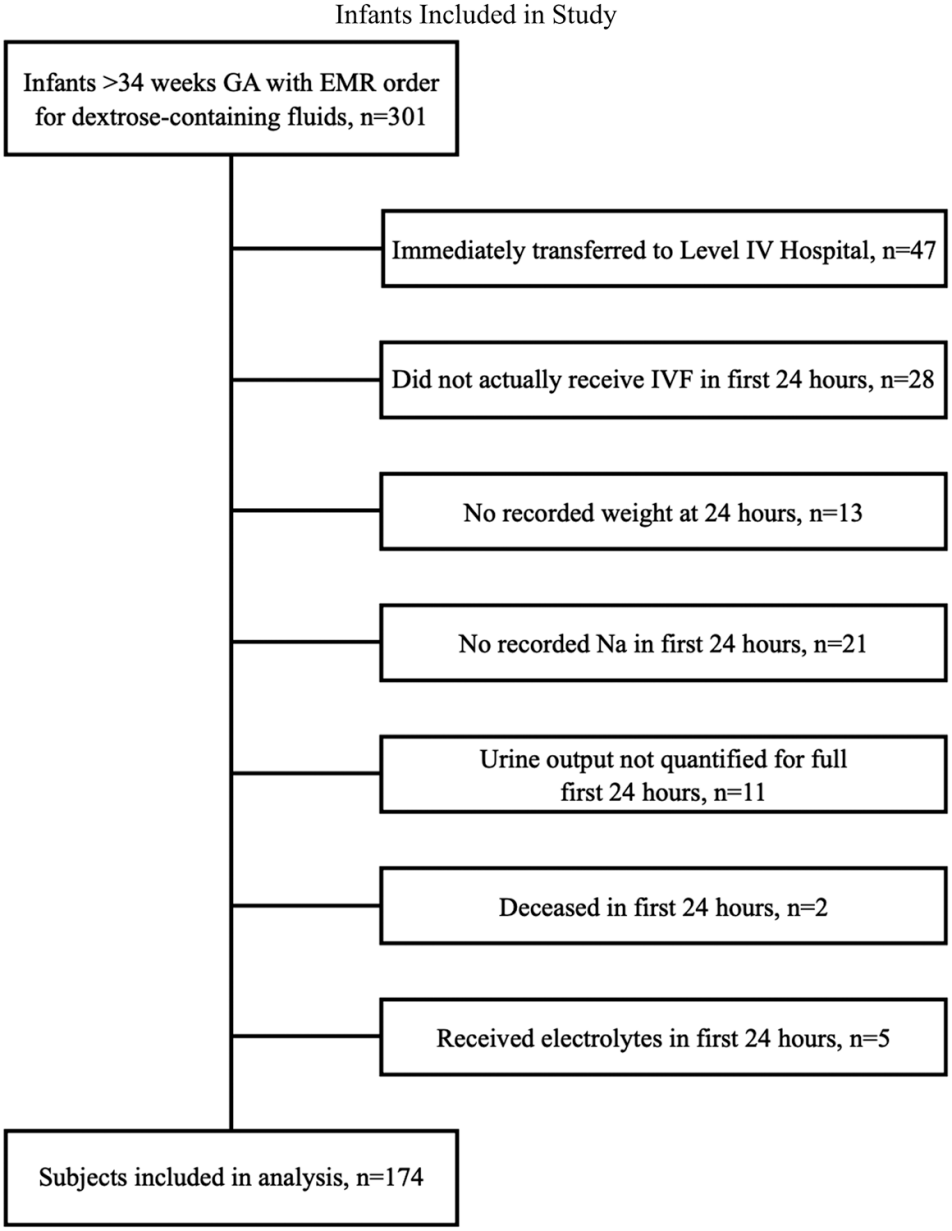
Flowchart for identification of infants meeting inclusion criteria. Abbreviations: EMR, electronic medical record; GA, gestational age; IVF, intravenous fluid.

A total of 301 patients were included in the initial data frame. After excluding 47 infants transferred to our partner level IV hospital for known congenital anomaly, 28 infants who did not receive IVF, 13 infants with no recorded weight at 24 hours, 21 infants with no recorded sodium at 24 hours, 11 infants without strict urine output recorded, 5 infants who received IVF with electrolytes before 24 hours, and 2 infants who died before 24 hours of life, 174 patients were included in the analysis (Figure 1). Of the included newborns, the mean gestational age was 36.8 weeks (SD, 2.3; range, 34.0–41.3 weeks), with 94 (54%) babies born late preterm (Table 1). The infant population was predominantly white (60%) and not Hispanic and/or Latino (77%). The most common diagnoses on admission were prematurity (54%), respiratory distress (71%), rule-out sepsis (74%), and hypoglycemia (21%). Of the infants receiving respiratory support, 13 required ventilatory support (7.4%), 84 required continuous positive airway pressure (CPAP) (48.2%), 6 received nasal cannula greater than 2 L but less than CPAP (3.4%), and 21 received nasal cannula 2 L or less (12%). It is the unit’s practice to provide humidity on all respiratory support greater than 2 L.

### Outcomes

In the first 24 hours, mean IVF received was 57.2 mL/kg/d (SD, 14.91), 128 (74%) infants gained weight or had no weight change, 130 (75%) had positive FB, and 73 received no enteral feeds (42%). In infants who received enteral feeds, the mean enteral intake in the first 24 hours was 19.4 mL/kg/d (SD, 18.5; only formula or expressed breast milk included). Ninety-seven percent of infants received dextrose 10% in water for maintenance IVF, with the rest receiving dextrose 12.5% in water. The median amount of time on IVF was 2.4 days (IQR 1.5–3.8). The mean urine output in the first 24 hours was 2.2 mL/kg/h (SD, 1.1), with 29 (17%) infants having a urine output of less than 1.0 mL/kg/h in the first 24 hours.

**FIGURE 2.**
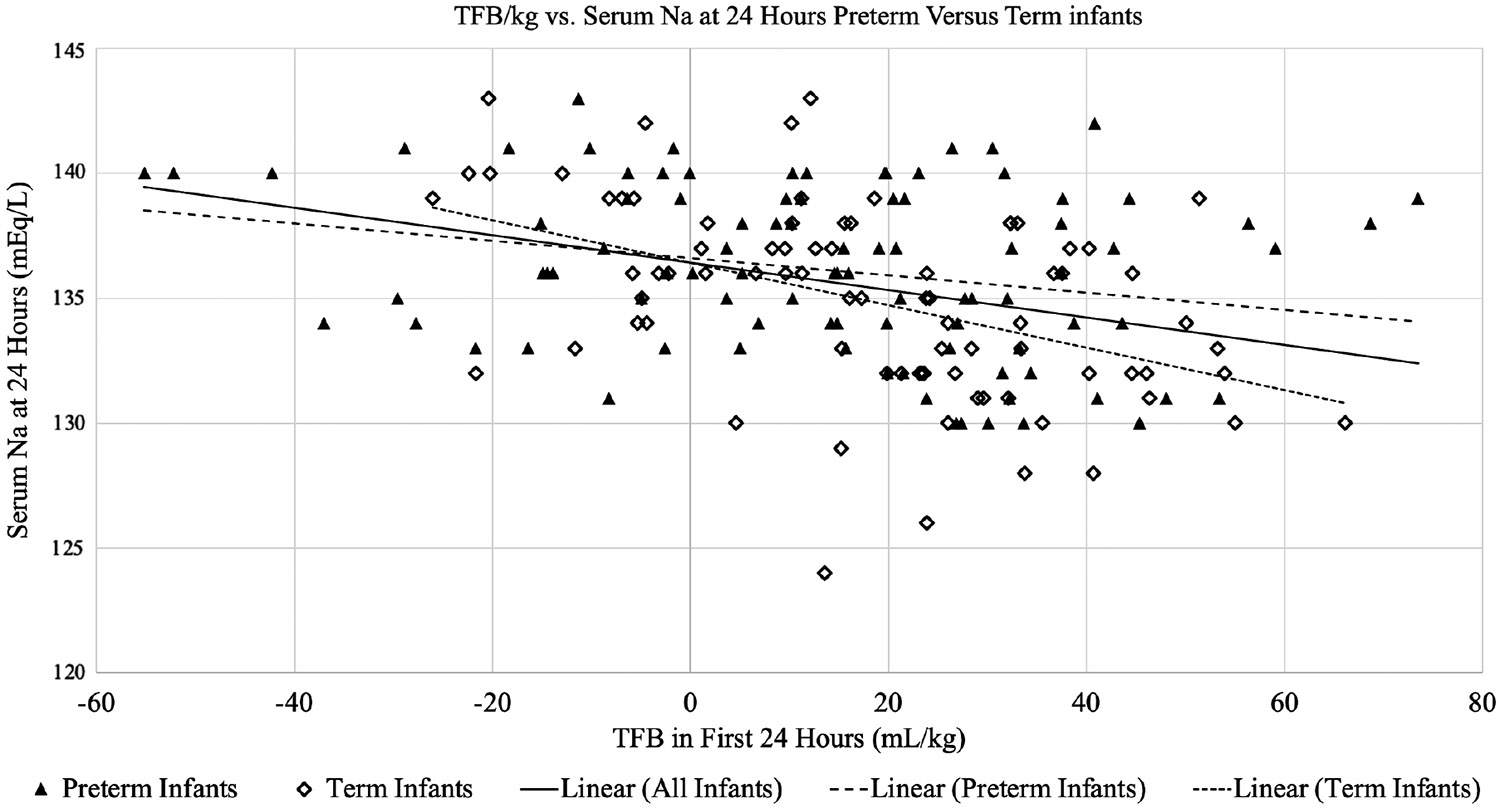
Preterm vs term infants: TFB per kilogram and 24-hour sodium. Abbreviation: TFB, total fluid balance.

The 24-hour serum sodium levels for our study population ranged from 124 to 143 mEq/L (mean, 135.5; SD, 3.7). Sixty-eight (39%) had sodium 134 mEq/dL or less, and 41 (24%) had sodium 132 mEq/dL or less. At 24 hours, term infants were more prone to sodium 132 mEq/dL or less (31% vs 17%; odds ratio [OR], 2.22; 95% CI, 1.08–4.54; *P* =.03) but not sodium 134 mEq/dL or less (45% vs 34%; OR, 1.58; 95% CI, 0.86–2.92; *P* =.14). Weight gain and FB were strongly correlated, and both were associated with low sodium. A multivariate regression model showed a 0.07-mEq/L decrease in serum sodium (95% CI, −0.09 to −0.05; *P* < .001) and a 6% increase in odds of sodium 132 mEq/L or less (95% CI, 1.03–1.08; *P* < .001) for every milliliter per kilogram of positive FB at 24 hours. Term infants showed decreases in sodium of 0.085 mEq/L/mL positive FB (95% CI, −0.12 to −0.047 mEq/L; *P* < .001) compared with 0.035 mEq/L/mL positive FB (95% CI, −0.06 to −0.007 mEq/L; *P* =.01) in preterm infants. In a multiple linear regression, patients who had positive FB exhibited sodium levels that were, on average, 2.49 U lower than those who had negative FB (*P* < .001), controlling for term gestation status (Figure 2).

**FIGURE 3.**
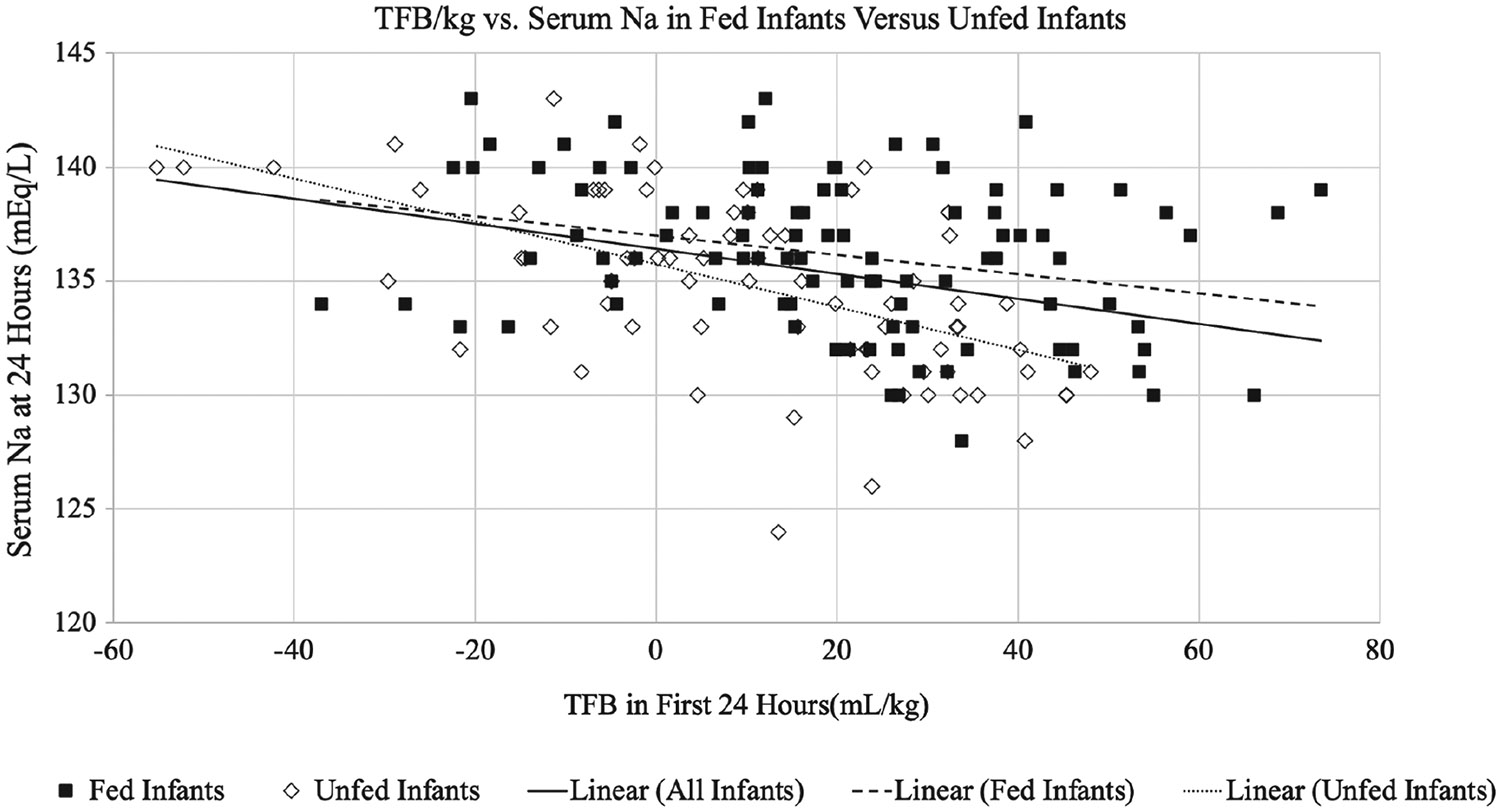
Fed vs unfed infants: TFB per kilogram and 24-hour sodium. Abbreviation: TFB, total fluid balance.

In a follow-up analysis of fed vs unfed infants, we observed a decrease of 0.094 mEq/L in serum sodium status for infants who received no feeds (95% CI, −0.13 to −0.06 mEq/L; *P* < .001) compared with a decrease in sodium of 0.040 mEq/L/mL positive FB (95% CI, −0.07 to −0.01 mEq/L; *P* =.006) in infants fed any amount in the first 24 hours. Logistic regression analysis indicated that for every 1-U increase in FB, there was a 7% increase in the odds of sodium levels being 132 mEq/L or less for unfed infants (OR, 1.07; 95% CI, 1.03–1.11; *P* < .001) compared with a 5% increase in the odds of sodium levels being 132 mEq/L or less for infants fed any amount (95% CI, 1.02–1.08; *P* =.002). These associations remained significant after adjusting for respiratory support, mode of delivery, and antibiotic use (Figure 3).

## Discussion

In our population of late preterm and term neonates on fluid compositions and volumes commonly initiated in many units, nearly 40% of infants had sodium levels outside the recommended range, 24% had sodium levels 132 mEq/L or less, over 70% had failure to lose weight at 24 hours, and almost 75% had positive FB. We found that a model incorporating urine output to calculate overall FB showed substantial correlation with serum sodium levels and weight change, which was particularly pronounced in term infants and those not enterally fed. Our study echoed prior studies in finding no significant association between amount of IVF (mL/kg) given and serum sodium when urine output was not considered.^22^ Our findings were also consistent with the findings of the multicenter Assessment of Worldwide Acute Kidney Injury Epidemiology in Neonates study in which weight gain and positive FB were also seen in over 65% of term and near-term infants on fluids at standard rates.^23^

Data on hypotonic fluid administration among near-term and term infants are lacking, despite strong, emerging evidence of risks among older children. Our small study suggests that hypotonic IVF use at current recommended volumes may contribute to hyponatremia when a positive FB and weight gain are seen at 24 hours. Although unit practices vary, many guidelines continue to recommend fluid for near-term and term infants based on milliliters per kilogram, increasing the fluid between day 1 and 2 of life regardless of weight change or urine output and withholding electrolytes until 24 hours of life.^7-10^ Furthermore, many units recommend initiation of fluids at 60 ml/kg/d, a much higher volume than the spontaneously feeding term infant would receive.^24^

Prior studies have demonstrated the detrimental effects of fluid overload in the neonatal and pediatric populations, including impaired oxygenation, acute kidney injury, need for mechanical ventilation, increased morbidity, and increased length of stay.^23,25-29^ Hyponatremia is one of the most common electrolyte abnormalities in neonates and is often iatrogenic.^30^ Severe dysnatremia in hospitalized children and neonates has been associated with the development of seizures, hearing loss, acute kidney injury, and increased morbidity and mortality.^31-38^ Small studies have also shown an association of sodium 132 mEq/L or less with adverse long-term outcomes in infants aged less than 34 weeks.^39,40^ Our study suggests that near-term and term infants, who comprise the majority of NICU admissions worldwide, may also be at risk of fluid overload and hyponatremia with current fluid guidelines.

Our study found that the effect of positive FB on hyponatremia was more pronounced in infants who did not receive any enteral feeds. The benefits of early initiation of minimal enteral nutrition are well demonstrated among critically ill infants and include promotion of intestinal motility, maturation, improved feeding tolerance, reduced risk of sepsis, and decreased time to achieving full enteral feeds.^41-43^ As we highlight here, an additional benefit may include improved sodium and FB in the early postnatal period. Animal models have shown that enteral feeding stimulates a complex neuroendocrine cascade that promotes diuresis.^44-46^ Need for respiratory support and use of antibiotics did not substantially alter our results, but our study was small and we were unable to adjust for other clinical factors. Larger studies are needed to explore this relationship.

To our knowledge, our study is the first to incorporate total fluid intake, urine output, and sodium concentration with standard IVF administration rates in late preterm and term neonates. Our findings of weight gain, positive FB, and hyponatremia with typical IVF rates used in many NICUs must be interpreted with caution because our study is limited by small sample size in a single-center, retrospective nature and had an inability to quantify volume of breastfeeding for total fluid balance, compare different IVF administration strategies, and fully adjust for clinical status. In addition, because our delivery hospital only has a level III NICU, infants with antenatally diagnosed complex congenital anomalies are transferred to our partner level IV hospital after birth and were therefore excluded from our study. We also excluded infants who were initially admitted to our newborn nursery, where urine output is not quantified. As a result, our study may be biased toward babies that are moderately ill. Routine laboratories are not obtained in infants who do not receive IVF. Thus, we were unable to examine a control group. Finally, although our study examines the impact of positive FB on serum sodium levels at 24 hours, unit practice after the first 24 hours is not standardized, and we were unable to examine the association between positive FB and any morbidity or mortality measures in our population.

This study highlights the need for further study of fluid management in near-term and term infants. Our data suggest that current practices of fluid initiation may hinder natural newborn physiologic weight loss and diuresis essential for successful transition to the extrauterine environment, particularly in term and unfed infants. The development of a dynamic fluid adjustment algorithm incorporating FB, electrolytes, and early feeding warrants further study and may be advantageous over conventional recommendations for this population. Larger trials examining the effects of a dynamic fluid approach and other fluid initiation choices are necessary for determining the optimal management of neonatal fluid needs in late preterm and term infants, who continue to comprise the majority of NICU admissions worldwide.

## Figures and Tables

**TABLE 1.** Background Characteristics of Study Cohort

Characteristics	Late Preterm Infants (GA 34–37 weeks), N = 94	Term Infants (GA ≥37 weeks), N = 80	*P* Value
GA, median (IQR), weeks	34.5 (34.0–35.5)	39.1 (37.9–39.9)	<.001
Birthweight, median (IQR), grams	2283 (1965–2595)	3285 (2910–3752)	<.001
Cesarean born, n (%)	53 (56)	28 (35)	.008
Respiratory support, n (%)	66 (70)	58 (73)	.57
Intubation, n	7	6	
CPAP or nasal cannula ≥2 L, n	45	45	
Nasal cannula ≤2 L, n	14	7	
No respiratory support, n	28	22	
Received antibiotics, n (%)	59 (63)	61 (76)	.08
Received no feeds first 24 h, n (%)	45 (48)	51(64)	.05
IVF intake, mean (SD), ml/kg/d	64.1 (13.6)	48.9 (11.9)	<.001
No weight loss at 24 h, n (%)	73 (78)	55 (69)	.25
Fluid balance, mean (SD), ml/kg/d	14.13 (25.2)	18.44 (20.7)	.23
Positive fluid balance, *n* (%)	67 (71)	63 (79)	.34
Sodium at 24 h, median (IQR), mEq/L	136 (134–139)	135 (132–138)	.03
Sodium ≤132 mEq/L at 24 h, n (%)	16 (17)	25 (31)	.03
Sodium ≤134 mEq/L at 24 h, n (%)	32 (34)	36 (45)	.14
CGA at discharge, mean (SD), days	36.9 (1.0)	39.7 (1.2)	<.001

Abbreviations: CGA, corrected gestational age; CPAP, continuous positive airway pressure; GA, gestational age; IVF, intravenous fluid.

## WHAT’S KNOWN ON THIS SUBJECT:

The use of hypotonic fluids in infants has been called into question, and use of these fluids is no longer recommended in infants older than 28 days. There are little data on fluid management in late preterm and term neonates.

## WHAT THIS STUDY ADDS:

Use of hypotonic fluids in late preterm and term infants at standard rates is associated with hyponatremia and weight gain, particularly in term infants. Enteral feeding mitigates this effect. Current fluid strategies may overestimate needs, particularly in term, unfed infants.

## Abbreviations

CPAP: continuous positive airway pressure
FB: fluid balance
IVF: intravenous fluid
NICU: neonatal intensive care unit
OR: odds ratio
REDCap: Research Electronic Data Capture
